# Adverse Behavioral Changes in Adult Mice Following Neonatal Repeated Exposure to Pain and Sucrose

**DOI:** 10.3389/fpsyg.2018.02394

**Published:** 2019-01-21

**Authors:** Manon Ranger, Sophie Tremblay, Cecil M. Y. Chau, Liisa Holsti, Ruth E. Grunau, Daniel Goldowitz

**Affiliations:** ^1^School of Nursing, The University of British Columbia, Vancouver, BC, Canada; ^2^BC Children’s Hospital Research Institute, Vancouver, BC, Canada; ^3^CHU Ste-Justine Research Centre, Montreal, QC, Canada; ^4^Centre for Molecular Medicine and Therapeutics, Vancouver, BC, Canada; ^5^Department of Occupational Science and Occupational Therapy, The University of British Columbia, Vancouver, BC, Canada; ^6^Department of Pediatrics, The University of British Columbia, Vancouver, BC, Canada

**Keywords:** sucrose, pain, prematurity, NICU, mouse model, neurodevelopmental outcomes

## Abstract

Sucrose is recommended for the treatment of pain during minor procedures in preterm infants in the neonatal intensive care unit (NICU) and is currently used worldwide as the standard of care. We recently reported that adult mice repetitively exposed to sucrose compared to water during the first week of life, irrespective of exposure to an intervention, had significantly smaller brain volumes in large white matter, cortical and subcortical structures (e.g., hippocampus, striatum, fimbria). These structures are important for stress regulation and memory formation. Here, we report the effects of repeated neonatal exposure to pain and sucrose on adult behavior in mice. Neonatal C57BL/6J mice (*N* = 160, 47% male) were randomly assigned to one of two treatments (sucrose, water) and one of three interventions (needle-prick, tactile, handling). Pups received 10 interventions daily from postnatal day 1 (P1) to P6. A single dose of 24% sucrose or water was given orally 2 min before each intervention. At adulthood (P60-85) mice underwent behavioral testing to assess spatial memory, anxiety, motor function, pain sensitivity, and sugar preference. We found that mice that had received sucrose and handling only, had poorer short-term memory in adulthood compared to water/handling controls (*p* < 0.05). When exposed to pain, mice treated with repetitive sucrose or water did not differ on memory performance (*p* = 0.1). A sugar preference test showed that adult mice that received sucrose before an intervention as pups consumed less sugar solution compared to controls or those that received water before pain (*p* < 0.05). There were no significant group differences in anxiety, motor, or pain sensitivity. In a mouse model that closely mimics NICU care, we show for the first time that memory in adulthood was poorer for mice exposed to pain during the first week of life, irrespective of sucrose treatment, suggesting that sucrose does not protect memory performance when administered for pain. In the absence of pain, early repetitive sucrose exposure induced poorer short-term memory, highlighting the importance of accurate pain assessment.

## Introduction

An estimated 15 million infants are born preterm (<37 weeks gestational age [GA]) each year according to a recent report by the World Health Organization, and this number is rising (World Health Organization, 2018). In North America this represents ∼8% of all live births, of these about 1% are born very preterm (24–32 weeks GA) 2 to 4 months early (Hamilton et al., 2015). Although the survival rate has increased substantially, over one-quarter of surviving infants experience moderate to severe neurodevelopmental problems, including poor motor and cognitive outcomes (Kuban et al., 2016; Heeren et al., 2017). Very preterm birth is coupled with an array of significant early-life stressors such as maternal separation, as well as exposure to pain, inflammation and pharmacological treatments. During their extended stay in the neonatal intensive care unit (NICU), very preterm infants undergo ∼200 painful procedures (Grunau et al., 2007), averaging 10 invasive and stressful procedures per day (Roofthooft et al., 2014). The negative effects of early untreated pain on brain development and behavorial outcomes have been demonstrated in both rodents (Anand et al., 1999; Dührsen et al., 2013), and humans (reviewed in Ranger and Grunau, 2014; Vinall and Grunau, 2014). Our longitudinal cohort studies in humans found short-term (Brummelte et al., 2012; Zwicker et al., 2013) and long-term adverse (Ranger et al., 2013, 2014, 2015; Vinall et al., 2014) effects of repetitive exposure to early pain-related stress on brain development and neurodevelopmental outcomes in preterm children, after accounting for clinical risk factors related to prematurity.

Effective pain management is essential to help mitigate these negative consequences of early pain exposure in very preterm children. However, the optimal strategy to achieve this goal is unclear. Oral sucrose, known as a non-pharmacological agent, is now used worldwide as the standard of care in neonatal units to alleviate acute procedural pain, but its safety relative to neurobehavioral outcomes remains to be determined (Gao et al., 2016; Stevens et al., 2016). To date, only one clinical study (albeit short term) has examined neurodevelopment after repeated sucrose administration in very preterm infants. Johnston and colleagues found that in infants born below 31 weeks GA, more than 10 sucrose doses per day given in the first week of life was associated with poorer attention and motor function at term-equivalent age (Johnston et al., 2007). Effects of repetitive sucrose on longer-term neurobehavioral development has not been studied, and this lack of safety data was highlighted in two recent reviews (Gao et al., 2016; Stevens et al., 2016). There is a growing concern regarding the use of sucrose in this population. Recently, the American Academy of Pediatrics cautioned use of sucrose for infant pain management until appropriate dose, mechanisms of action, and long-term effects of this treatment are addressed; sucrose should be viewed as a prescribed medication that must be tracked (Committee on Fetus and Newborn and Section on Anesthesiology and Pain Medicine, 2016).

Mechanisms of sucrose-induced analgesia are well-established in rodent models. Oral administration of sweet substances such as sucrose inhibits pain by mediating endogenous opioid peptide and μ1-opioid receptor actions (de Freitas et al., 2012). Others have suggested additional involvement of the 5-HT2A-serotonergic receptors in the antinociception effect of sweet solution administration (Rebouças et al., 2005). Key brainstem sites critically involved in descending pain modulation have been shown to be activited by intraoral sucrose administration in neonatal rats (Anseloni et al., 2005). Studies in adult rodents have shown that repeated sucrose doses lead to higher levels of the neurotransmitters dopamine and acetylcholine (Hajnal et al., 2004; Spangler et al., 2004; Rada et al., 2005). Dopamine plays a key role in motor and cognitive functions (Dreisbach et al., 2005), and acetylcholine in attention, memory, learning, and pain (Lagercrantz et al., 2010). In the developing preterm brain, it is not known whether increases in dopamine and/or acetylcholine levels would have positive or negative effects, either short or long-term, on related functions (Holsti and Grunau, 2010).

To our knowledge, only two pre-clinical studies have examined the effects of early repetitive exposure to pain and/or sucrose on adult memory (Nuseir et al., 2015, 2017). However, in one of those studies, neonatal sucrose and pain exposure were induced over far longer periods than would be developmentally relevant as a model of preterm NICU care (Nuseir et al., 2015). Using a mouse model of pain and sucrose administration which closely mimics the exposure of preterm infants in the NICU, we previously reported widespread long-term alterations in white and gray matter brain volumes in adult mice repeatedly exposed to sucrose compared to water in the first week of life (Tremblay et al., 2017b). In that study, irrespective of pain exposure, repetitive sucrose induced smaller brain volumes mainly in white matter regions of the forebrain, cerebellum, and hippocampus. Consistent with our findings in mice, in human preterm infants, higher exposure to glucose for pain relief in the NICU was associated with lower thalamic volume on neonatal MRI (Schneider et al., 2018). There appear to be no animal studies of effects of neonatal repeated sucrose in the context of pain on neurobehavior functions such as anxiety, motor, and cognition, that accurately models the duration and frequency of exposure in humans following preterm birth.

Given that sucrose treatment is currently administered to thousands of preterm infants for minor procedural pain relief, it is crucial to determine the long-term consequences of repetitive sucrose for pain management on neurodevelopment. Therefore, we examined effects of neonatal repetitive sucrose exposure on behavioral and cognitive outcomes in adulthood in a mouse model that closely mimics pain of minor procedures during NICU care.

## Materials and Methods

### Animals

All animal procedures were approved by The University of British Columbia Animal Care Committee and conform to the guidelines outlined by the Canadian Council on Animal Care. Animals were maintained on a 14/10 h light/dark cycle with food and water *ad libitum*. Mice were provided with nestlets and Plexiglas igloo-style houses as part of standard enrichment. Cellulose bedding was used to minimize discomfort of inflamed paws in the pups (1/4-inch pelleted cellulose; Biofresh). C57BL/6J and ICR (CD1) mice used in this study were obtained from the Goldowitz Laboratory mouse inbred and outbred colonies, respectively. A non-nursing ICR female mouse was added to the litter to prevent rejection of pups from nursing C57BL/6J dam and improve survival rate of treated mouse pups (Tremblay et al., 2017a). Postnatal day 0 (P0) was defined as the day of birth. Pups were left with their nursing dam and non-nursing ICR female until the age of weaning, on P21. After weaning, mice were ear-notched to allow for the identification of individual mice and housed together with appropriate nesting materials, enrichment, and cellulose bedding (5 mice/cage, sex-matched cages) during aging and behavioral testing (study endpoint P85-P95).

### Experimental Design

On the day of birth (P0) newborn mouse pups were randomized to one of six groups; each litter included at least two different groups and nearly equal distribution by sex per group. For individual identification during infancy, pups were tattooed on their paw using a 30G needle prick. Experiments were started on the following day (P1) and continued until P6. Pups received either sterile water or sucrose 24% (Vehicle; Treatment) given orally by a micropipette 2 min before one of three interventions: paw needle-prick, light paw tactile pressure with a cotton-tipped swab (Tactile), or only being handled by simply picking up the pup (Handling). Treatment/Intervention sequence duration was less than 5 min per interval. Treatment/Intervention was administered 10 times per day per mouse pup over a 10-h period (from 8 AM until 6 PM) during the day (light) cycle from P1 to P6 inclusive. Each Treatment/Intervention was spaced by a minimum of 30 min to allow enough time for feeding, mother-pups interaction/care, and recovery from the interventions.

**FIGURE 1:**
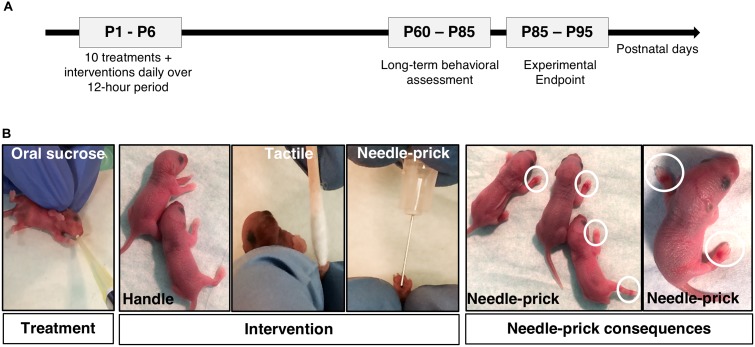
Neonatal mouse model of repetitive sucrose exposure given for procedural pain [adapted from Tremblay et al. (2017b)]. **(A)** Schematic illustrating the experimental protocol timeline. Long-term behavioral testing were performed between postnatal day (P) 60–85 following neonatal treatment/intervention period. **(B)** Images showing C57Bl/6J mouse pups receiving oral sucrose or water treatment preceding 10 daily interventions: Handle, Tactile, or Needle-prick. White circles are highlighting ecchymosis and inflamed paws seen on pups after needle-prick intervention. P, postnatal day.

The treatment consisted of a solution of sucrose 24% w/v [Sucrose ≥ 99.5% (GC) Sigma, United States, cat#S7903] in sterile water filtered with 0.22 μm filter, or sterile water, administered intraorally 2 min before the intervention. The treatment was administered either on the anterior part of the tongue or inner-cheek of the mouth using a micropipette and sterile tips. The dose of sucrose was based on the standard guideline recommended dose for human neonates, which is 0.5 mL per dose for very preterm infants (24 to 32 weeks of gestation), corresponding to 0.08–0.2 g of sucrose per body weight (kg) (Stevens et al., 2016). Pups received 0.1–0.2 g of sucrose per kg of body weight and the dose was adjusted daily based on the pups’ weights (i.e., ∼1 to 4 μl/dose during the course of the experiment). Mouse pups in the vehicle treatment groups received an equivalent volume of oral sterile water. For each intervention, the forepaws and hindpaws were alternated and only one paw was touched (tactile) or pricked. Needle-pricks were performed using a 30G sterile needle (0.3 mm outer diameter) angled at 10–15 degrees from the skin to carefully pierce only the surface of the skin, with special attention in avoiding penetration of deeper layers, such as tendon or bone. Mouse pups were returned to the dams between each hourly Treatment/Intervention sequence. To minimize heat loss by pups, all procedures were conducted on a circulating water heating pad. Prior to each procedure, C57BL/6J nursing females and ICR non-nursing females were transferred to a clean cage and housed in a separate room to prevent possible stress induced by sight, sound or smell from the procedures. At P7, pups remained in their home cage with their dam until weaning (P21). A total of 160 mouse pups (47% male) were treated and survived to adulthood with an overall survival rate of 93% (non-survival due to cannibalism, rejection of dam, and/or poor feeding during the first 2 days of life). Mice were weighed daily from P1 to P7, at weaning on P21, at the start and end of behavioral testing (P60, P80-85), and finally at experimental protocol endpoint (P85-95). The timeline of the experimental design is shown in Figure 1A.

At P0, mouse pups (75 males; 85 females) were randomly allocated to one of six groups: Vehicle/Handling (*n* = 27), Vehicle/Tactile (*n* = 25), Vehicle/Needle-prick (*n* = 26), Sucrose/Handling (*n* = 27), Sucrose/Tactile (*n* = 28), or Sucrose/Needle-prick (*n* = 27) (Table 1). During the first days of life, from P1 to P3, pups in the needle-prick groups showed visible paw inflammation at the site of skin-breaks as exposure to the intervention accumulated throughout the 10 h. In each case, local inflammation disappeared by the following morning. No signs of infection or other complications occurred (Figure 1B).

**Table 1:** Study sample group allocation and weight trajectory.

Treatment pre-		*Mean weight gain during*	*Mean body weight at*		Overall distribution
intervention	Intervention	*treatment (P7 weight–P1 weight)*	*behavioral testing*	*n (%Males)*	(*N* = 160)
Water	Handling	2.1 ± 0.1 g	21.3 ± 0.6 g	27 (44.4%)	*N* = 78
Water	Tactile	2.1 ± 0.1 g	21.9 ± 0.7 g	25 (56.2%)	
Water	Needle-prick	1.9 ± 0.1 g	21.9 ± 0.7 g	26 (50.0%)	
Sucrose 24%	Handling	1.8 ± 0.1 g	21.6 ± 0.6 g	27 (44.4%)	*N* = 82
Sucrose 24%	Tactile	1.8 ± 0.1 g	21.2 ± 0.6 g	28 (42.9%)	
Sucrose 24%	Needle-prick	1.9 ± 0.1 g	22.2 ± 0.7 g	27 (38.9%)	


*Neonatal mice (*n* = 160) were assigned randomly across treatment/intervention groups (total of six groups). Table provides the distribution/sex ratio, mean weight gain during treatment period, and mean body weight at behavioral testing. Values are means ± SEM. P, postnatal day.*

### Behavioral Evaluation During Adulthood

Behavior testing took place when adult mice reached P60 and lasted 2 to 3 weeks (endpoint ∼P80-85). The mice were transferred to the behavioral testing suite 2 weeks before testing to acclimate to the room. All testing sessions were conducted during the light cycle, between 7 AM to 6 PM. All groups went through the same non-randomized behavioral testing sequence: Open-Field, Elevated Plus Maze, Morris Water Maze, Rotarod, Hot Plate, and Sugar Preference. Males and females were tested separately; female rats were tested first, followed by the males.

#### General Locomotor Activity Assessment

The Open-Field test was used to assess locomotor activity, anxiety and behavior in a novel environment (Gould et al., 2009). A plexiglass chamber of 50 cm × 50 cm × 12 cm was used. Individual adult mice were placed into the chamber and allowed to explore the environment for 10 min while locomotion was recorded by a camera placed directly above the chamber and analyzed by an image tracking system (Noldus Ethovision XT software; Noldus Information Technology, United States). Measures included total distance traveled, percent time spent in movement and mean velocity.

#### Anxiety-Like Behavior Assessment

The Elevated Plus Maze was used to assess general anxiety behavior (Balsevich et al., 2014). A maze was placed 55 cm above floor with four black Plexiglas arms, two open arms (67 cm × 7 cm) and two enclosed arms (67 cm × 7 cm × 17 cm) which formed a cross shape opposing each open area. Adult mice were placed in the center of the maze and allowed to explore for 5 min. A camera mounted directly above the maze recorded the behavior of the animal and was analyzed by the image tracking system (Noldus Ethovision XT software; Noldus Information Technology, United States). Measurements included time spent in open and enclosed arms and number of entries in open areas.

#### Gross Motor Function, Balance, and Coordination

The Rotarod test was used to measure balance, motor coordination, and motor learning on an accelerating rotarod (Ugo Basile, Italy) (Buitrago et al., 2004). Mice were placed on a rotating rod in individual compartments (up to five mice during one session); distance from the rotating rod to the base of the apparatus was approximately 10 cm. The rotation speed of the rotating rod was accelerated from 4 to 40 rpm over 5 min. Each trial lasted a maximum of 300 s or when the mouse fell off the rotating rod. The duration of time spent on the rotating rod was recorded for each trial. Trials were conducted over 3 days including five trials on the first day followed by three trials per day with a minimum of 30-min inter-trial. After each completed trial, the mice were returned to their home cage.

#### Spatial Learning and Memory Assessment

Spatial learning and memory abilities were tested by the Morris water maze test (Morris, 1984; Vorhees and Williams, 2006; Hamre et al., 2007). Latency to find the platform was recorded manually as well as quantified using an automated video tracking system located above the pool and analyzed with Noldus Ethovision XT 7.0 software (Noldus Information Technology, United States). The pool was filled with room temperature water (1 m in depth) and made opaque with white non-toxic water-soluble paint. A platform was located in an arbitrarily defined quadrant of the maze and 2 cm below the surface of the water so that mice could not see the platform when swimming. Mice were trained for 3 days to assess learning (2 tests/day- in AM and PM for 3 days) and two probe trials in the absence of the platform were used to test short (on fourth day) and long-term (7 days post-training) memory (Tremblay et al., 2017a). Between each session, mice were resting in a heated standard cage with paper towel and water gel for a 10 min inter-trial period. In each of the trials, mice were given 60 s to locate the platform. Probe trials were performed without the platform and mice were recorded over a 60 s period prior to being removed from the pool. Measures included a learning curve over 3 days, distance covered and latency to reach the hidden platform, average swim speed and percent of time spent in their respective platform quadrant. Manually recorded measures of latency to find platform during the 3-day training period were used in our analysis to assess learning, all other reported measures were quantified using the automated video tracking system (Noldus EthovisionXT7.0 Software).

#### Pain Threshold

A Hot-plate test for pain threshold was performed with an analgesiometer. The apparatus consists of 25 by 25 cm metal hot-plate surface set at 52°C, a Plexiglas cage to restrain the animal fitting over the hot plate, and a foot-switch operated timer. Pain threshold was measured by the latency to nociceptive responses (paw lift, limb shaking, or paw lick) with a maximum cut-off time of 30 s to avoid any tissue injury. Latency to pain was calculated from averaging the results from three trials spaced by 15 min between each trial. The surface temperature (i.e., 52°C) and 30 s cut-off time assured that none of the mice endured any kind of skin injury during this test.

#### Sugar Preference

At the end of the behavior testing period, mice were challenged with a sugar preference test (Bachmanov et al., 1996a,b; Avena et al., 2004). Mice were housed in single-plex cages (with bedding, housing and chow) with *ad libitum* access to two bottles containing 50 ml each of either water or 10% sucrose water placed side by side for 48 h (bottles were switched position at 24 h interval). Mice and chow were weighed pre- and post-sugar preference test; amount of liquid in each bottle was measured pre- and post-test. Percent of water versus 10% sucrose water consumed was calculated.

### Statistical Analysis

Group comparisons were carried out using analysis of variance (ANOVA) or MANOVA, repeated-measures ANOVA, Mauchly’s test, and Multivariate test Pillai’s Trace to examine differences for all neurobehavioral outcome measures (i.e., open field, elevated plus maze, Morris water maze, rotarod, hot plate, and sugar preference) across the three treatment and three intervention groups followed by Fisher’s least significant difference (LSD) or Tukey’s honestly significant difference (HSD) test *post hoc* tests, unless otherwise specified. Sex was also examined in our statistical models. Statistical analyses were performed using the Statistical Package for Social Sciences (SPSS) version 22 (IBM, Somers, NY, United States); *p*-values < 0.05 were considered statistically significant. Exclusion of outliers from behavioral and structural analyses was made prior to statistical analysis; outliers were defined by being below 1.5x the interquartile range from the 25^th^ percentile or above 1.5x the interquartile range from the 75^th^ percentile. Data were graphically organized using GraphPad Prism version 7.0 (San Diego, CA, United States). Data are presented graphically as means ± SEM.

## Results

### Repeated Pain and/or Sucrose Does Not Affect Weight Trajectory Throughout Life

Daily weights were captured from P1 to P7 and prior to behavioral testing at P60 (Table 1). One-way ANOVA analysis showed that mean weight gain during the treatment period, measured by the difference between P7 and P1 weights, was significantly different between groups (*F*_(5,154)_ = 2.825, *p* = 0.02). However, the significant difference between groups was not evident after correcting for multiple comparisons using Tukey’s HSD *post hoc* test. At adulthood prior to behavioral testing (P60), there were no significant differences in mean body weight between groups (*F*_(5,154)_ = 0.369, *p* = 0.87). No sex effect was found.

### Long-Term Behavioral Alterations of Adult Mice Exposed to Repetitive Sucrose and/or Interventions Over the Neonatal Period

#### Neonatal Repetitive Treatment and/or Intervention Exposure Did Not Alter General Locomotor Activity, Anxiety-Like Behaviors, or Gross Motor Function in Adult Mice

**FIGURE 2:**
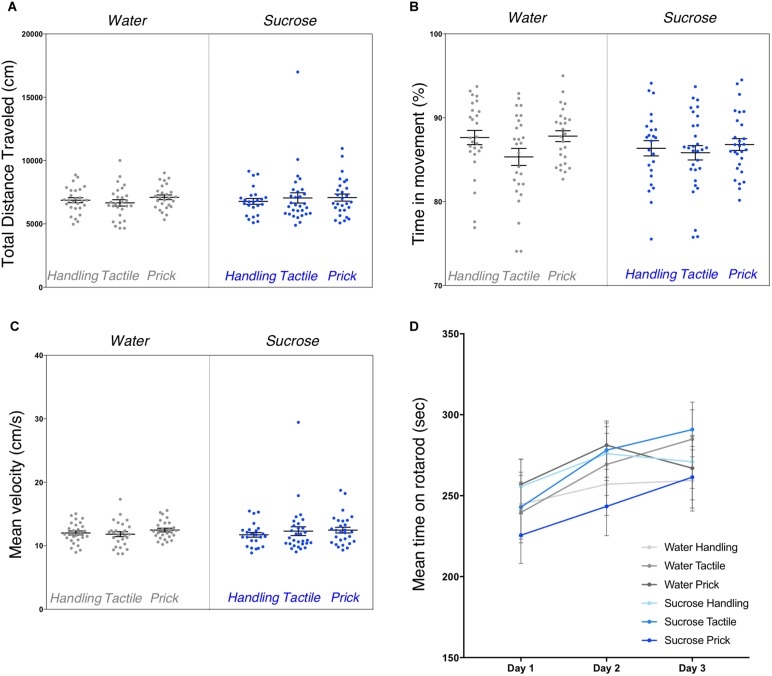
Assessment of general motor function and coordination after neonatal repetitive exposure to treatment prior to an intervention. Open-field testing performed to assess locomotion capacities in adult mice from six experimental groups. Box plots show the total distance traveled (*p* = 0.83) **(A)**, the percentage of time spent in movement (*p* = 0.23) **(B)** and mean velocities (*p* = 0.78) **(C)** measured between groups. Data presented as scatter plots with mean ± SEM; horizontal bars represent mean values; black asterisks and bars denote statistical significance using ANOVA. Graph in **(D)** shows the mean time adult mice spent on rotarod (i.e., latency to fall) learning curves between the six experimental groups. All groups learned to stay longer on the rod over the 3 days (*p* < 0.0001), but there were no significant differences in time to learn over days between experimental groups (*p* = 0.58), or interaction between trials and groups (*p* = 0.58). *n* = 24–27 per group.

Repeated exposure to sucrose and/or intervention did not have a significant effect on any of the general locomotor activity measures during the open-field test (Figures 2A–C). One-way ANOVA analyses did not show significant differences between groups for the total distance traveled during recording (*F*_(5,156)_ = 0.429, *p* = 0.83), the mean velocity (*F*_(5,156)_ = 0.493, *p* = 0.78) and the percentage of time spent in movement (*F*_(5,156)_ = 1.386, *p* = 0.23) (Figure 2). No sex effect was found.

Gross motor function assessed by the rotarod test revealed a highly significant trial effect on overall mean time spent on rotarod across the 3 days of training (repeated measure ANOVA multivariate test Pillai’s Trace *V* = 0.12, *F*_(2,153)_ = 10.45, *p* < 0.0001) (Figure 2D). Mauchly’s test indicated that the assumption of sphericity was violated (χ^2^_(2)_ = 25.8, *p* < 0.0001), multivariate (MANOVA) tests are reported (ε = 0.87) for within subject analysis. We did not find any significant effect of group (*F*_(5,154)_ = 0.76, *p* = 0.58) or interaction between trial/training and group (multivariate test Pillai’s trace *V* = 0.054, *F*_(10,308)_ = 0.85, *p* = 0.58). Here, we found that overall, adult mice showed improvement performance on the rod across trials, irrespective of group allocation. No sex effect was found.

**FIGURE 3:**
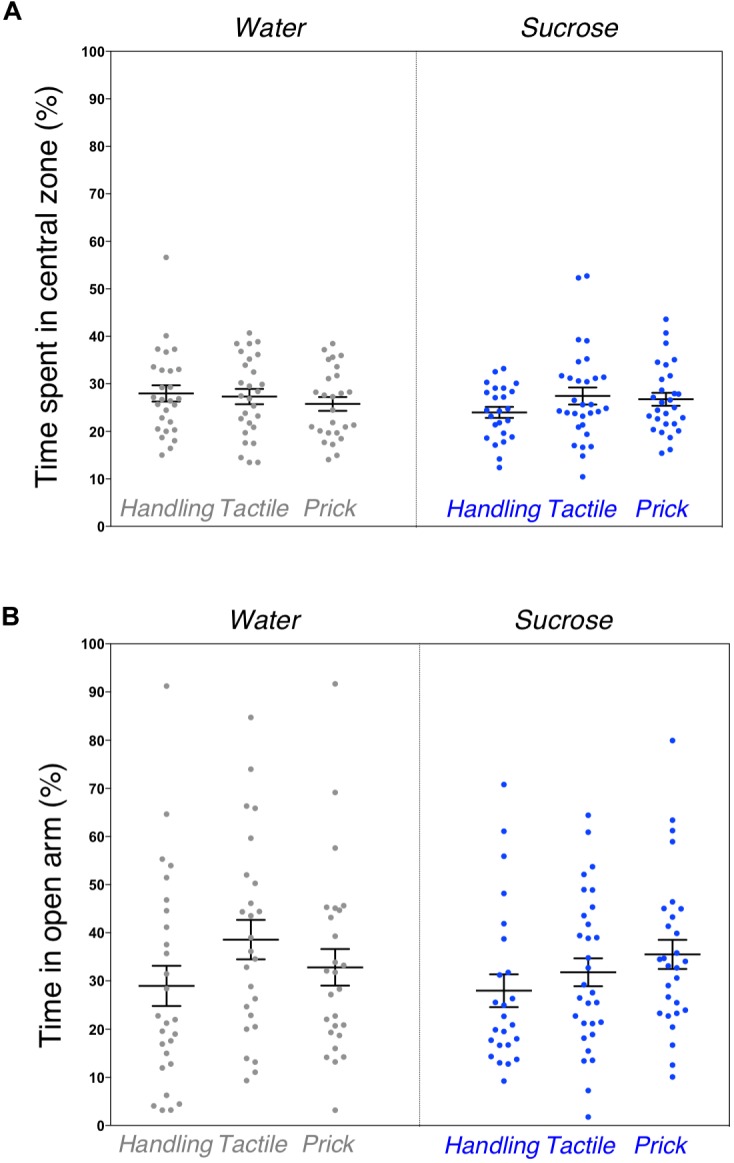
Assessment of anxious behavior after neonatal repeated exposure to treatment prior to an intervention. Box plots comparing six experimental groups **(A)** showing the time spent in central zone of a novel environment measured during the open field test (*p* = 0.55). **(B)** Percent of time spent in open arms of an elevated plus maze (*p* = 0.32). Data presented as scatter plots with mean ± SEM; horizontal bars represent mean values; black asterisks and bars denote statistical significance using ANOVA. *n* = 24–27 per group.

Similarly to the general locomotor assessment, exploratory and anxious behaviors in a novel environment examined by the elevated plus maze test revealed no significant difference between the six groups. The percentage of time spent in the central zone of the open-field arena (*F*_(5,156)_ = 0.821, *p* = 0.55) along with the amount of time spent in the open arm of the elevated plus maze (*F*_(5,154)_ = 1.187, *p* = 0.32) were not statistically different (Figures 3A,B). No sex effect was found.

#### Neonatal Repetitive Pain and Sucrose Impacts Short-Term Memory in Adulthood

**FIGURE 4:**
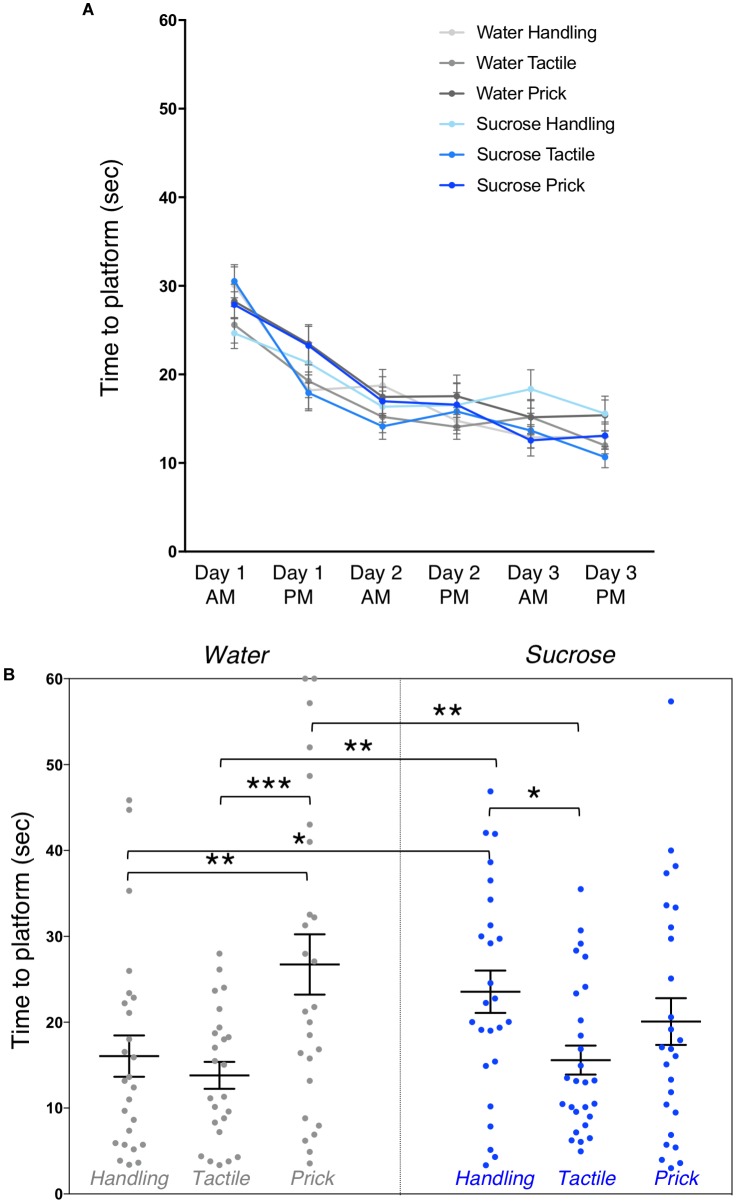
Effects of treatment and intervention on short-term memory in adulthood (Morris water maze test). **(A)** Training. Morris water maze learning curves are shown from testing day 1 to day 3. All groups learned to locate the platform over their six trials (*p* < 0.0001), but there were no significant differences in time to learn over days between experimental groups (*p* = 0.57), or interaction between trials and groups (*p* = 0.13). **(B)** Short-term memory. Group comparison of adult mice time to reach the area where the platform was during training during the first testing probe (1 day post-training). Compared to adult mice in the Water/Needle-prick group, mice in the Water/Handling (*p* = 0.003), Water/Tactile (*p <* 0.001) and Sucrose/Tactile (*p* = 0.002) groups took significantly less time to locate the “platform” on the MWM test. Compared to mice in the Sucrose/Handling group, those in the Water/Handling (*p* = 0.04) and Water/Tactile (*p* = 0.01) and Sucrose/Tactile (*p* = 0.02) took less time to locate the “platform.” Data presented as scatter plots with mean ± SEM; horizontal bars represent mean values; black asterisks and bars denote statistical significance using ANOVA. *n* = 24–27 per group. ^∗^*p* < 0.05; ^∗∗^*p* < 0.01; ^∗∗∗^*p* < 0.001.

Learning on the Morris water maze showed a highly significant trial effect on overall mean time to locate the platform across the 3 days of training (multivariate test Pillai’s trace *V* = 0.67, *F*_(5,143)_ = 56.93, *p* < 0.0001) (Figure 4A). Mauchly’s test indicated that the assumption of sphericity was violated (χ^2^_(14)_ = 85.6, *p* < 0.0001), multivariate (MANOVA) tests are reported (ε = 0.82) for within subject analysis. We did not find any significant effect of group (*F*_(5,143)_ = 0.77, *p* = 0.57) or interaction between trial and group (multivariate test Pillai’s trace *V* = 0.22, *F*_(25,735)_ = 1.33, *p* = 0.13) on learning. Thus, showing that overall, adult mice learned and improved their time to locate the platform across trials irrespective of group allocation. No sex effect was found.

Short-term memory during the probe test, 1 day after 3-days training, revealed an overall effect of group on time to reach the area where the platform was during training (*F*_(5,147)_ = 4.12, *p* = 0.002). *Post hoc* test, shown here as mean differences (confidence intervals), uncovered that mice in the Water/Needle-Prick group (pain) took significantly longer to reach the “platform” compared to those in the Water/Handling (10.68 [3.71, 17.65], *p* = 0.003), Water/Tactile (12.92 [5.87, 19.96], *p* < 0.001), and Sucrose/Tactile (11.14 [4.31, 17.98], *p* = 0.002) groups. Mice in the Sucrose/Handling group took significantly longer to locate the “platform” compared to Water/Handling (7.5 [0.46, 14.54], *p* = 0.04), Water/Tactile 9.74 [2.63, 16.85], *p* = 0.01), and Sucrose/Tactile (7.96 [1.06, 14.87], *p* = 0.02). Adult mice that received repetitive needle-pricks preceded by water or that received sucrose but were simply handled during the first week of life had poorer short-term memory at adulthood. There was no significant difference in the time to locate the “platform” between mice in the Water/Needle-prick and Sucrose/Needle-prick groups (*p* = 0.1) (Figure 4B). When exposed to needle-pricks, mice that were treated with repetitive sucrose or water did not differ on their short-term memory performance at adulthood. The significant effects of sucrose and/or pain on short-memory were no longer present at the second testing probe (i.e., long-term memory; *p* = 0.8). Male and female mice did not differ significantly in their short or long-term memory performances.

#### Sugar Preference Was Altered in Adults Exposed to Sucrose During the Neonatal Period

**FIGURE 5:**
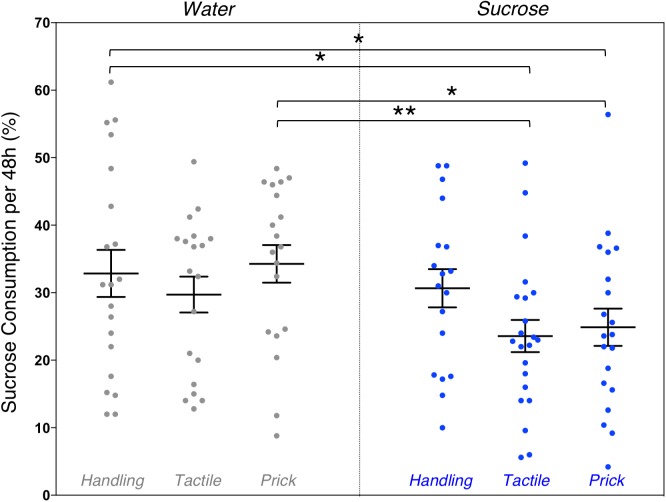
Effects of treatment and intervention on sugar preference test. Group comparisons of percent of 10% sucrose water consumed from the total liquid consumption during the 48 h sugar preference test. Compared to adult mice in the Water/Handling group (controls), those in the Sucrose/Tactile (*p* = 0.019) and Sucrose/Needle-prick (*p* = 0.047) groups consumed significantly less 10% sucrose water. Adult mice in the neonatal Sucrose/Tactile (*p* = 0.008) and Sucrose/Needle-prick (*p* = 0.021) groups consumed significantly less 10% sucrose water compared to those in the Water/Needle-prick group. Data presented as scatter plots with mean ± SEM; horizontal bars represent mean values; black asterisks and bars denote statistical significance using ANOVA. *n* = 18–22 per group. ^∗^*p* < 0.05; ^∗∗^*p* < 0.01.

Due to time constraints, the sugar preference test was conducted on a smaller sample of mice (*n* = 118). A one-way ANOVA revealed an overall significant group difference on percentage of 10% sucrose water consumed during the 48 h sugar preference test (*F*_(5,117)_ = 2.36, *p* = 0.044). LSD *post hoc* test group comparisons showed significant mean differences between Water/Handling (controls) compared to Sucrose/Tactile (-9.28 ml [1.58,16.97], *p* = 0.019) and Sucrose/Needle-prick (-7.97 ml [0.09, 15.85], *p* = 0.047) groups, as well as between Water/Needle-prick compared to Sucrose/Tactile (-10.7 ml [2.9, 18.5], *p* = 0.008) and Sucrose/Needle-prick (-9.39 ml [1.41, 17.37], *p* = 0.021) groups (Figure 5). Overall during the 48 h sugar preference, adult mice that were repetitively exposed to both an intervention (tactile or needle-prick) and treatment (sucrose) as pups consumed significantly less sucrose water compared to those that received water as treatment during the first week of life. Mice that were simply handled and exposed to sucrose in early-life did not significantly differ from any other group in the amount of sucrose water consumed at adulthood. No sex effect was found.

#### Pain Threshold Was Not Altered

**FIGURE 6:**
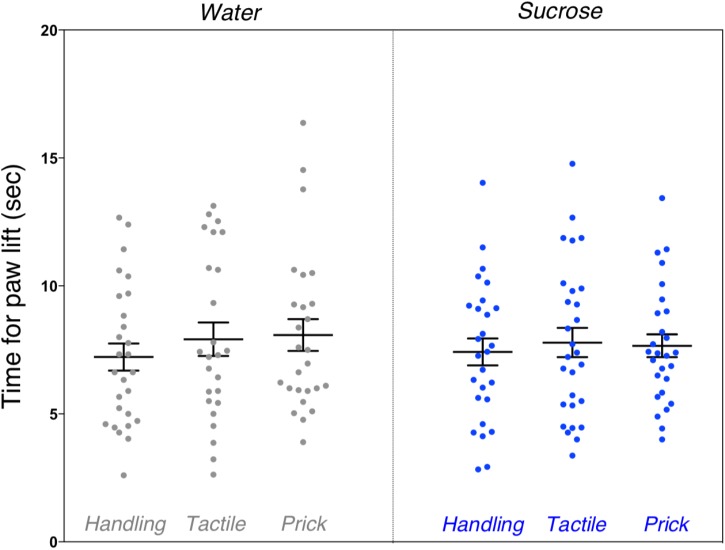
Assessment of pain threshold during hot plate test after neonatal repetitive exposure to treatment prior to an intervention. Adult mice’s mean time taken for paw lift in response to hot plate test indicative of pain threshold comparing each group. No significant difference was found between the six groups (*p* = 0.61). Data presented as scatter plots with mean ± SEM; horizontal bars represent mean values; black asterisks and bars denote statistical significance using ANOVA. *n* = 24–27 per group.

We found no significant differences between the groups on mean time for paw lift from hot plate (*F*_(5,154)_ = 0.72, *p* = 0.61). There was no difference in pain threshold between male and female mice (Figure 6).

## Discussion

This is the first study to examine the effects of early repetitive pain and sucrose exposure on long-term behavioral and cognitive outcomes using a model that closely mimics NICU pain and treatment of the very preterm neonate. Our key findings were that memory in adulthood was poorer for mice exposed to pain during the first week of life, regardless of sucrose treatment, suggesting that sucrose is not protective for memory performance when administered for pain. We also found that in the absence of pain, when mouse pups were only handled, early sucrose exposure induced poorer short-term memory in adulthood. Finally, sugar preference in adult mice was lower in those exposed to early sucrose before an intervention, indicating possible conditioned memory and anhedonic behavior.

These important findings on adult memory are consistent with our earlier findings that mouse pups exposed to neonatal repetitive sucrose had smaller adult brain volumes in important regions involved in memory formation (Tremblay et al., 2017b). Specifically, in that study, sucrose exposure during the first week of life induced smaller volumes of the hippocampus, dentate gyrus, and stratum granulosum of the hippocampus and the fornix along with other brain regions including cortical, subcortical gray and white matter structures in adulthood. These significant changes were found irrespective of the type of intervention (needle-prick, tactile, or handling) in the neonatal period consistent with the adult functional alterations affecting memory seen in our current study. Neurodevelopmental consequences of postnatal stress have been studied extensively in preclinical models. A recent review examining the developmental effects of early life stress exposure in mice, focusing on the maternal separation paradigm, found that the most robust effect was on memory performance (poorer spatial memory across all strains) (Tractenberg et al., 2016). In a longitudinal cohort study following very preterm infants exposed to oral glucose with limited sedative and analgesic medications for pain management, a higher number of invasive procedures was associated with slower growth of important brain structures such as the thalamus and basal ganglia (Schneider et al., 2018). Similar to the findings in our mouse model, glucose administration for procedural pain management did not mitigate the deleterious effect of procedural pain on the developing brain of these very preterm infants. Higher procedural pain and glucose exposure were associated with poorer psychomotor development at 18 months corrected age. This reinforces earlier findings by Johnston and colleagues that found that very preterm infants exposed to more than 10 doses of sucrose per day during the first week of life in the NICU showed poorer motor function at term-equivalent age (Johnston et al., 2007).

In our study, we assessed neurobehavioral changes in adulthood, but not earlier. We found gross motor function and coordination were not significantly affected. One explanation for the differences between effects of sucrose on human infant motor outcome at term-equivalent age and lack of such findings in our current study, may be that the damaging effect of repetitive exposure to sugar solutions on motor development may not persist into adulthood. In our previous work (Tremblay et al., 2017b), we found that adult mice exposed to sucrose as pups, irrespective of intervention exposure, had significantly smaller volumes in the cerebellum, specifically in the anterior lobules III–V, which are cerebellar regions involved primarily in motor control. We aimed here to measure a broad array of behaviors to examine the overall impact of early repetitive sucrose exposure. This broad strategy limited our ability to assess more detailed fine motor and cognitive functions, such as using the skilled walking assessment on the horizonatal ladder (Metz and Whishaw, 2002) or the beam walking assay (Southwell et al., 2009) for instance. It would be important to further examine the functional impact of neonatal repetitive sucrose exposure on cerebellar structures at various developemental ages in a rodent model.

Our sugar preference findings of reduced sucrose water solution consumption in mice exposed to early repetitive interventions and sucrose treatment support the established evidence that chronic mild stress exposure in rodent models of early-life adversity (e.g., pups reared with limited bedding and nesting) is associated with reduced preference for palatable food (i.e., sugar) (Remus et al., 2015; Bolton et al., 2018). Early-life stress-induced behavioral disturbances, ranging from hippocampus-dependent memory deficits to problems with experiencing pleasure (anhedonia) have been well-documented (reviewed in Bolton et al., 2017). In our study, we aimed to mimic the use of sucrose as a treatment for procedural pain by repetitively exposing mouse pups to sucrose prior to an intervention. We examined if this early exposure to repetitive sugar would induce preference or aversion to sweet taste. Given the evidence of sugar addiction after excessive exposure intake (Avena et al., 2008), we were expecting to find that mice exposed to early sucrose, irrespective of the intervention, would consume more sugar water during the sugar preference test, which turned out not to be the case. As noted above, we interpreted our findings as more related to exposure to early stress (tactile or pain) in combination with sucrose, rather than the isolated effect of one of those stressors. If we interpret our current findings through the lense of an early-life stress exposure model, sucrose does not appear to be providing protection against the deleterious effects of early stress-related adversity (i.e., repetitve pain).

Assessing memory in rodents to reflect a cognitive process as complex as memory function in the human can be done by tapping into analogous brain regions. As such, in animal models, much of the research has focused on hippocampus-dependent memory, due to the availability of well-established standardized tests, such as the Morris water maze test which evaluates spatial navigation and memory (Rice and Barone, 2000), and well-characterized neural and molecular mechanisms (Bolton et al., 2017). We now have evidence that early repetitive exposure to both pain and sucrose has deleterious effects on both hippocampal volumes and hippocampus-dependent processes. Indeed, functionally, we showed in adulthood poorer short-term memory, as well as a reduced ability to experience pleasure (i.e., decreased consumption of sucrose water solution – anhedonia). Current understanding points to the involvement of posterior cerebellar regions (lobule VI, crus I, and crus II) in complex cognitive and memory operations in humans, such as working memory and spatial processing (Stoodley and Schmahmann, 2009). In our previous work, we found evidence of smaller volumes in posterior cerebellar subregions lobule VI and Crus I in adult mice previously exposed during the first week of life to sucrose before an intervention, which pertains to our current functional findings of poorer short-term memory (Tremblay et al., 2017b). The progressive stabilization of long-term memory after information acquisition is referred to as consolidation (Dudai, 2004). Two types of processes are commonly described: synaptic consolidation, which starts within a few minutes after the acquisition and lasts for hours in all memory systems and is hippocampal-dependent; and system consolidation, which takes longer to consolidate and will reorganize memories in a hippocampal-independent process (Dudai, 2004). The formation of short versus long-term memory relies on different neurological pathways (Schimanski et al., 2002) and may explain why we did not find any significant effects of either early repetitive pain and/or sucrose exposure on long-term memory (i.e., system consolidation). In mice, intact hippocampal structures (especially hippocampal commissure) and corpus callosum are necessary in order to perform well on short-term memory tasks, which is not essential for long-term memory consolidation (Schimanski et al., 2002). Supporting this evidence is our previously reported findings of altered volumes in both hippocampal structures and corpus callosum in adult mice exposed to sucrose as pups (Tremblay et al., 2017b).

Contrary to our findings, Nuseir and colleagues reported recently beneficial effects of repetitive exposure to sucrose treatment before needle pricks (4 times/day from P1-14) compared to pain alone in newborn male rats on pain sensitivity and long-term memory (probe test at 24 h) outcomes (Nuseir et al., 2017). Previous work from this same group showed similar protective effects of sucrose pre-treatment on pain thresholds and long-term memory compared to exposure to pain only, following 8 weeks of daily pain and/or sucrose exposure (Nuseir et al., 2015). In this particular study treatments lasted far beyond period analogous to NICU intensive care of very preterm neonates. In contrast, we used a pain model that much more closely matches that of the human infant exposure in the NICU. Moreover, there are a number of important differences between their two study models and ours, which may explain our discrepant findings and make it difficult to compare the outcomes. They used much higher sucrose dosing (0.2 ml of sucrose 25%); whereas, we administered a dose 100 times lower adjusted to daily weights. They used a far more invasive painful stimulus (25G needle inserted through the paw while we used a superficial prick with a 30G needle). Finally, they did not use a vehicle control treatment (i.e., water solution) and used rats rather than mice.

In rodent models of neonatal pain, specific patterns of long-term behavioral effects from exposure to repetitive acute pain from needle-pricks or severe inflammatory pain from formalin injections in the first week of life of rat pups have been demonstrated, such as decreased locomotor activity (Bhutta et al., 2001), increased anxiety and defensive withdrawal behavior (Anand et al., 1999), and social hypervigilance (Anand et al., 1999). We expected that mice exposed to needle-pricks during the first week of life would have altered motor and anxiety-like behaviors, which was not the case. Again, the species (mice versus rat) and methodological differences may account for the differences in findings. That is, inflammatory pain or pain from injections may not have the same consequences as superficial needle-pricks. Our relatively innocuous needle-prick pain stimulus is an important difference, given that we used very thin needles (30G) and did not penetrate deeper layers, such as tendon or bone, in contrast to the more invasive procedures used in needle-prick pain in previous rat studies (Anand et al., 1999; Davis et al., 2018).

Differences in our experiemental design regarding the delivery of the skin-breaking stimulus also may influence findings. We undertook interventions 10 times daily for 6 days, based on the reported median exposure to painful procedures during NICU care (Roofthooft et al., 2014). Parallel to our findings on long-term memory impairment of early pain exposure, Henderson and colleagues showed that even a single injection of formalin in the hindpaw of infant rats at birth altered hippocampal-dependent memory in adulthood (Henderson et al., 2015). A study in rat pups exposed to four daily hindpaw needle-pricks (24G) or slight touch during the first 7 days of life showed effects on fear conditioning (auditory freezing only) at post-weaning, adolescence, and adulthood ages (i.e., Ps 24, 45, 66) (Davis et al., 2018). Similar to our findings, they failed to show an effect on long-term sensory thresholds unless rats were exposed to fear conditioning prior to testing. Indeed, re-exposing rats to a stressful condition seems to be required to induce a mechanical hypersensitivity at P27 only (not at P48 or 69) and this in both touched and pricked rats. Thus, since we did not perform a pain re-exposure in adulthood, these latter findings may explain why we did not find any significant differences in pain thresholds at adulthood between groups in our study.

Our recent findings of adverse effects of pain and sucrose on adult regional brain volumes (Tremblay et al., 2017b) and behavioral outcomes in adult mice reported here may be related to specific neurotransmitter system alterations. Sucrose, with its opioid-like effect (Spangler et al., 2004), may be acting through the mesolimbic system (i.e., reward system in the brain). It has been established that dopamine neurons respond to aversive and/or rewarding stimuli, consequentally some neurons may be releasing dopamine in response to both punishing (e.g., pain) and rewarding (e.g., sucrose) stimulants (Bromberg-Martin et al., 2010). In this context, dopamine released in the mesolimbic system can modulate the salience of pain stimuli (Taylor et al., 2016), that is, tuning into the associated reward of pain relief. It is possible that if sucrose and pain are repetitively given in combination during a critical period of brain development, this “double-hit” of nearly constant dopamine release could eventually have detrimental effects on the brain regions involved and consequently affect motivation/reward-like behaviors. Given that sucrose is currently being administered to thousands of very preterm infants for procedural pain managent in NICUs worldwide, it is imperative to conduct further research to investigate the implications of these exposures on the mesolimbic dopaminergic and reward/motivation systems by using animal models.

A potentially important difference in our studies was the addition of a non-nursing ICR female mouse to the litter to support the dam and improve survival of mouse pups. The ICR female likely added a buffering effect on pain through increased grooming and nurturing of the mouse pups during this early-life stress exposure. Maternal behavior (grooming and licking) has been shown to modulate effects of early pain exposure in rats (Walker et al., 2003; de Medeiros et al., 2009), where increased maternal behaviors reduced inflammation in response to neonatal formalin injections during the first 2 weeks of life of rat pups and thermal sensitivity in adulthood. Strong evidence in both pre-clinical and human studies emphasizes the importance of early life sensory stimulation embedded in mother-infant nurturing interactions for shaping neurodevelopment (Myers et al., 2015; Welch et al., 2015, 2017; reviewed in Curley and Champagne, 2016). Importantly here, we did not find any protective effect of sucrose on pain since adult mice that received early repetitive painful stimuli irrespective of receiving water or sucrose as pre-treatment had similar long-term behavioral outcomes. The additional “maternal care” from the ICR non-nursing mouse may have been more powerful than sucrose at not only helping survival of our pups but also at alleviating the pain-related stress. Similarly, naturally occurring variations in maternal rearing behaviors in rodents impact neurobehavioral development, the stress-response system and gene expression (as reviewed in Curley and Champagne, 2016; Bedrosian et al., 2018); low compared to high licking/grooming mothers during the postnatal period results in poorer neurodevelopment in the offspring. Communal nesting, whereby several lactating females care for a pooled group of offspring, which is similar to our current model, has demonstrated comparable beneficial developmental effects on pups (as reviewed in Curley and Champagne, 2016). The focus of our current study was not to examine maternal rearing. Given that we did not monitor maternal behaviors of dam and ICR mouse, we can only speculate on the possible buffering effect of our communal nurturing set-up. Additonal research is needed to examine these interactions and possible mitigating effects of maternal care.

## Conclusion

Based on our current and previous findings using the same mouse model of early repetitive pain and sucrose exposure, we have shown that sucrose was not protective for long-term adverse effects of procedural pain on both brain development and behavioral outcomes such as memory function. Most importantly, in the absence of pain, neonatal sucrose seems to negatively impact brain volumes and adversely affect adult memory. Animal research needs to be interpreted with caution and cannot be directly applied to humans. Nonetheless, given the world-wide use of sucrose for procedural pain treatment in the preterm population and the growing evidence in regards to possible detrimental effects of repetitve sucrose exposure during a period of developmental vulnerability of preterm infants, cautious use of this standard of care procedural pain management strategy in this fragile population is advocated. More clinical longitudinal studies assessing the long-term effects of early exposure to sucrose are urgently needed. Current studies show that human touch-based treatments (e.g., skin-to-skin, facilitated tucking, maternal touch) appear to protect brain development (Milgrom et al., 2010; Myers et al., 2015; Olsson et al., 2016; Maitre et al., 2017), therefore may be better alternatives for treatment of procedural pain in preterm infants.

## Author Contributions

MR, ST, LH, RG, and DG participated in conception, design of research, and interpreted results of experiments. MR and ST performed the experiments, prepared the figures and tables, and drafted the manuscript. MR, ST, and CC analyzed the data. All authors edited and revised the manuscript and approved the final version of manuscript.

## Conflict of Interest Statement

LH is a lead inventor of a medical device for pain management for preterm infants, for which she could receive remunerations in the future. The remaining authors declare that the research was conducted in the absence of any commercial or financial relationships that could be construed as a potential conflict of interest.
